# Emerging Risk of Flea-Borne *Bartonella* in Tropical Cities: Evidence from Stray Cats in the Klang Valley, Malaysia

**DOI:** 10.3390/insects16121282

**Published:** 2025-12-18

**Authors:** Justin Titti Alison, Auni Atikah AbdulHalim, Muhammad Rasul Abdullah Halim, Hasmawati Yahaya, Muhamad Afiq Aziz, Sazaly AbuBakar, Zubaidah Ya’cob

**Affiliations:** 1 Higher Institution Centre of Excellence (HICoE), Tropical Infectious Diseases Research & Education Centre (TIDREC), Universiti Malaya, Kuala Lumpur 50603, Malaysia; 2 Department of Ecology and Biodiversity, Institute of Biological Sciences, Faculty of Science, Universiti Malaya, Kuala Lumpur 50603, Malaysia; 3 Department of Microbiology, Institute of Biological Sciences, Faculty of Science, Universiti Malaya, Kuala Lumpur 50603, Malaysia

**Keywords:** vector, zoonosis, *Felis catus*, infectious disease, animal management

## Abstract

Urban stray cats are often associated with ectoparasites and zoonotic pathogens due to their unsanitary living conditions and lack of veterinary care. Fleas, especially *Ctenocephalides* spp., are competent vectors of *Bartonella* spp., a genus of emerging bacterial pathogens with both public health and veterinary relevance. This study investigated the presence of *Bartonella* DNA in ectoparasitic fleas infesting stray cats in various urban habitats within the Klang Valley, Malaysia. A total of 204 fleas were collected from 89 stray cats. Fleas were identified morphologically using established taxonomic keys under a light microscope and further validated through PCR amplification of the mitochondrial cytochrome *c* oxidase subunit I (*cox1*) gene. Detection of *Bartonella* spp. was conducted by targeting the citrate synthase (*gltA*) gene. All fleas were confirmed as *Ctenocephalides felis*, with an infestation prevalence of 39.33% among the cats sampled. Of 118 *C. felis* specimens tested, 86.44% were positive for *Bartonella* DNA, one of the highest worldwide and significantly surpassing previous Malaysian reports. Sequencing of 12 positive samples showed identities with *B. claridgeiae* (58.3%), *B. henselae* (25.0%), an uncultured *Bartonella* species (8.3%) and a *Bartonella* isolate from a dog in Chile (8.3%). These results highlight the significant presence of *Bartonella*, causative agent of cat-scratch disease in stray cats, emphasizing their potential role as urban reservoirs and vectors. The findings underscore the need for ectoparasite surveillance and zoonotic pathogen control as integral components of stray animal management in Malaysia’s urban settings.

## Simple Summary

Stray cats living in cities often carry fleas, which can spread pathogens that cause diseases to humans and animals. In this study, we examined fleas collected from stray cats in different sites within Klang Valley, Malaysia, to find out if they carried any harmful pathogen. We first confirmed that all the fleas collected were cat fleas (*Ctenocephalides felis*), the most common flea that parasitize cats. We then tested the fleas for the presence of *Bartonella* bacteria, which can cause “Cat Scratch Disease (CSD)” in humans. Our results showed a very high number of infected fleas. These findings show that there may be a hidden health risk to communities living in close contact with stray cats, especially in crowded city areas. This study highlights the importance of monitoring fleas and the bacteria they carry as part of public health efforts. By understanding these risks early, health authorities and communities can take steps to manage stray animals and prevent the spread of diseases to humans.

## 1. Introduction

Stray cats are ubiquitous in urban ecosystems, often living in close proximity to humans and other domestic animals [1]. Many of these cats originate from abandoned pets or unplanned litters, forming unregulated populations that thrive in densely populated areas [2]. Due to limited access to veterinary care and poor health management, stray cats are frequently infested with ectoparasites, which play a key role in maintaining and transmitting zoonotic pathogens of public health significance [3]. This human–animal interface, intensified by urbanisation, provides an ideal setting for the emergence and spillover of vector-borne infections under the One Health framework.

Among feline ectoparasites, fleas, particularly the cat flea *Ctenocephalides felis*, are the most common and globally widespread [4]. *C. felis* exhibits remarkable ecological adaptability, infesting a wide range of mammalian hosts across both temperate and tropical regions [5]. Beyond causing dermatological irritation and allergic reactions in animals, *C. felis* serves as a competent vector for several zoonotic agents, notably *Bartonella* species. Of these, *B. henselae* and *B. clarridgeiae* are of major medical relevance, being the primary etiological agents of cat scratch disease (CSD) in humans [6]. Human infection typically results from scratches or bites contaminated with flea faeces [7], and while CSD is usually self-limiting in immunocompetent individuals [8], severe systemic manifestations such as osteomyelitis, arthritis and involvement of the central nervous system have been documented, particularly in immunocompromised patients [9]. Human exposure to *Bartonella* is widespread, with seroprevalence reaching nearly 28% [10]. The global burden of this disease is considerable, with estimated incidence rates of 6.4 cases per 100,000 individuals among adults and 9.4 cases per 100,000 individuals in children aged 5–9 years [11].

Globally, urban stray cats are increasingly recognised as reservoirs for *Bartonella* spp. and their flea vectors [12]. Reported infection rates of *Bartonella* species in cat populations vary considerably by location, ranging from 0% to 62% globally [13,14] and reaching even higher levels in isolated cat populations [15]. In Malaysia, however, studies on flea infestations and *Bartonella* infections in stray cats remain limited, often constrained by small sample sizes or narrow geographic coverage.

Although animal-based data are limited, emerging evidence from Malaysian health facilities suggests that *Bartonella* exposure in humans may be under-recognized. A serological review of febrile patients from a major teaching hospital in the Klang Valley detected *Bartonella* IgG antibodies in 21.6% of individuals tested, including those initially suspected of rickettsial or dengue infections, highlighting that *Bartonella* exposure among humans in this region may be more common than previously recognised [16]. Additionally, a clinical review of Malaysian bartonellosis cases documented 19 patients diagnosed with ocular bartonellosis over a three-year period, representing one of the few documented series of human *Bartonella* infections in the country [17]. Notably, only 26.3% of these patients recalled cat scratches or bites, indicating that a substantial proportion of infections may arise from unrecognised or indirect exposures [17]. These findings underscore the public health relevance of flea-borne *Bartonella* in metropolitan Malaysian settings and reinforce the need for epidemiological data from both reservoirs and clinical populations.

The Klang Valley, encompassing Kuala Lumpur and its surrounding metropolitan districts, is one of the most densely populated and urbanised regions in the country, hosting a large and growing population of free-roaming cats. Despite the frequent human–cat interactions in this region, data on the prevalence of *C. felis* infestations and their role as vectors of *Bartonella* spp. are scarce [6,18,19]. This paucity of epidemiological evidence limits public health risk assessment and hinders the development of evidence-based vector control strategies.

The present study aims to address this knowledge gap by investigating the natural infection rates of *C. felis* and *Bartonella* spp. in urban stray cats within the Klang Valley, Peninsular Malaysia. By integrating morphological and molecular identification approaches, this research provides updated insights into flea-borne *Bartonella* circulation in urban environments. The findings are expected to contribute to the growing body of One Health evidence needed to inform zoonotic disease surveillance, risk mitigation, and integrated vector management strategies in tropical urban settings.

## 2. Materials and Methods

### 2.1. Study Area and Flea Collection

**Figure 1:**
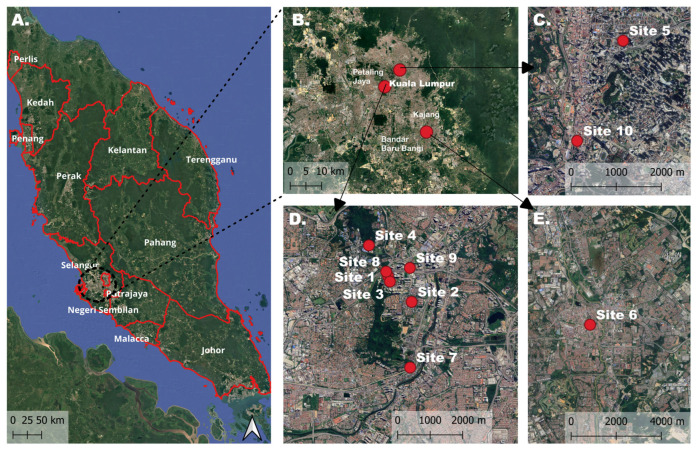
Maps illustrating the distributions of the sampling sites for stray cat screening in Klang Valley, Peninsular Malaysia. (**A**) Peninsular Malaysia; (**B**) Sampling sites within Klang Valley; (**C**) Sites 5 and 10 in Kampung Baru and Pasar Seni, Kuala Lumpur; (**D**) Sites 1–4 and 7–9 around Pantai Dalam and Bangsar South, Kuala Lumpur and Petaling Jaya, Selangor; (**E**) Site 6 in Kajang, Selangor.

All procedures involving animal handling and sample collection were conducted in strict accordance with institutional and international ethical standards for the humane treatment of animals. Approval was obtained from the Institutional Animal Care and Use Committee (IACUC), Universiti Malaya (Ethics Reference No: T/22052023/13032023-02/R). Flea sampling was conducted daily from 8:00 am until 5.30 pm between October and December 2023 across ten urban sites within the Klang Valley, Peninsular Malaysia and are mapped using QGIS (version 3.28) (Figure 1, Table 1). This region represents one of the most densely populated and urbanised areas of the country, characterised by a large stray cat population living in close proximity to humans. Each site was visited only once to avoid resampling of individual cats. The number of sampling sites varied among location types to reflect differences in free-roaming cat availability and accessibility. Housing areas and public transportation hubs each had three feasible sites, while eatery areas had four sites due to higher cat activity and ease of access. Site selection also considered ethical and safety requirements, as well as permissions from local authorities, ensuring that only locations suitable for humane, low-stress sampling were included.

Free-roaming cats encountered at the selected urban and suburban locations within the Klang Valley were first visually assessed from a distance to ensure they exhibited no signs of illness, severe injury, or aggressive behaviour. Only cats that appeared alert, ambulatory, and behaviourally calm were considered eligible for sampling. To minimize stress, cats were approached slowly using small food pellets as bait to encourage them to remain stationary. Once the cat demonstrated relaxed feeding behaviour, a gentle manual restraint technique was applied, involving minimal physical contact and avoiding forceful handling. No sedation, chemical immobilisation, or capture cages were used at any stage. Ectoparasite collection was performed using a fine-toothed stainless-steel flea comb. Combing was conducted systematically for approximately 2–3 min per cat, focusing on anatomical regions known to harbour fleas, including the head, neck, dorsal torso, ventral abdomen, hindquarters, and tail base. Each combing pass was inspected visually. Immediately after each combing session, the comb containing dislodged fleas was inserted into a sterile zip-lock bag and sealed to prevent escape. The sealed bag was gently shaken to detach fleas from the comb’s teeth before transferring the fleas into labelled collection tubes containing 70% ethanol for preservation. Personal protective equipment (PPE), including nitrile gloves and long-sleeved laboratory coats, was worn throughout the procedure to minimise the risk of scratches, bites, and zoonotic exposure. All sampling equipment was disinfected with 70% ethanol between animals to avoid cross-contamination. Upon completion of flea collection, each cat was released at the exact location where it was initially encountered. No animal was restrained for more than a few minutes, and all individuals were released unharmed.

All fleas were transported to the laboratory and preserved at −80 °C until further processing and each flea was subjected to morphological identification performed under a NIKON stereomicroscope (SMZ800N, (Tokyo, Japan)) using standard morphological identification keys [20].

### 2.2. Flea Genomic DNA Extraction

For molecular analysis, a maximum of 10 fleas per infested cat were randomly selected to provide a representative subset while avoiding overrepresentation from heavily infested individuals, as commonly applied in ectoparasite pathogen studies [21,22]. DNA was extracted from each flea using standard genomic extraction protocols. All remaining fleas were preserved at −80 °C for potential future analyses. In brief, genomic DNA was extracted from 118 individual fleas using the DNeasy Blood and Tissue Kit (Qiagen, Hilden, Germany) following the manufacturer’s protocol, with minor modifications. Each flea was homogenized in 180 µL of ATL buffer using a sterile micropestle (Chongqing New World Trading Co., Ltd., Chongqing, China) followed by the addition of 20 µL of Proteinase K. The samples were incubated at 56 °C with intermittent vortexing until complete tissue digestion was achieved. Subsequently, 200 µL of AL buffer and 200 µL of absolute ethanol were added to each lysate, which was then transferred to a spin column and centrifuged at 6000× *g* for 1 min. The column was washed sequentially with 500 µL of AW1 and AW2 buffers. Finally, DNA was eluted in 50 µL of AE buffer and stored at −20 °C until polymerase chain reaction (PCR) analysis.

### 2.3. Molecular Identification of Fleas

Molecular confirmation of flea species was performed via PCR amplification of the mitochondrial cytochrome *c* oxidase subunit I (*cox1*) gene using the primer pair LCO and Cff-R [23] (Table 2), yielding an expected amplicon size of approximately 550 bp. Each 25 µL PCR reaction contained 12.5 µL of DreamTaq Green PCR Master Mix (Thermo Scientific, Vilnius, Lithuania), 1 µL of each primer (10 µM), 2 µL of genomic DNA, and 8.5 µL of nuclease-free water. Amplification was carried out under the following thermocycling conditions: initial denaturation at 95 °C for 1 min, followed by 35 cycles of denaturation at 95 °C for 15 s, annealing at 55 °C for 15 s, and extension at 72 °C for 10 s, with a final extension at 72 °C for 5 min (Table 3). Positive controls (known *C. felis* DNA) and negative controls (nuclease-free water) were included in each PCR run. Amplicons were visualized by electrophoresis on 1.0% agarose gels stained with GelRed and compared against a 100 bp GeneRuler DNA ladder (Thermo Fisher, Waltham, MA, USA). Positive PCR products were purified and sent for sequencing (Apical Scientific Sdn. Bhd., Seri Kembangan, Malaysia) for species confirmation.

### 2.4. Detection of Bartonella spp. By PCR

Screening for *Bartonella* DNA was performed by amplifying a fragment of the citrate synthase (*gltA*) gene using the primer pair BhCS.781p (5′-GGGGACCAGCTCATGGTGG-3′) and BhCS.1137n (5′-AATGCAAAAAGAACAGTAAACA-3′) as described by Norman et al. [24] (Table 2), generating a 379 bp amplicon. Each PCR reaction (25 µL) contained 12.5 µL of DreamTaq Green PCR Master Mix (Thermo Scientific, Vilnius, Lithuania), 1 µL of each primer (10 µM), 2 µL of flea genomic DNA, and 8.5 µL of nuclease-free water. Thermocycling conditions consisted of an initial denaturation at 94 °C for 10 min, followed by 35 cycles of denaturation at 94 °C for 30 s, annealing at 51 °C for 45 s, and extension at 72 °C for 30 s, with a final extension at 72 °C for 7 min (Table 3). Positive and negative controls were included in all PCR assays, using *Bartonella henselae* DNA as the positive control and sterile nuclease-free water as the negative control. PCR products were visualized via electrophoresis on 1.0% agarose gels stained with GelRed and compared to a 100 bp molecular size marker. Positive amplicons were purified and submitted for bidirectional sequencing at Apical Scientific Sdn. Bhd. (Seri Kembangan, Selangor, Malaysia).

### 2.5. Sequence Alignment and Phylogenetic Analysis

Raw chromatograms were examined using SeqScanner v2 (Applied Biosystems, Waltham, MA, USA) and manually edited for base-calling accuracy in BioEdit v7. Sequences were aligned using MEGA v11 [25] with default parameters, and consensus sequences were compared against the NCBI GenBank database using BLASTn via the online NCBI platform for species identification. Phylogenetic trees were constructed using the Maximum Likelihood (ML) method in MEGA v11. The Tamura 3-parameter (T92) model was applied for *cox1* gene sequences of fleas, while the Kimura 2-parameter (K2) model was used for *Bartonella gltA* sequences. Node reliability was evaluated with 1000 bootstrap replicates. *Pulex irritans* [26] and *Brucella melitensis* [27] were designated as outgroups for flea and *Bartonella* phylogenies, respectively. Reference sequences retrieved from GenBank were included to confirm genetic clustering and species assignment.

### 2.6. Flea Infestation Parameters and Statistical Analysis

Flea infestation parameters were quantified following the definitions proposed by [28] to provide a comprehensive assessment of parasite–host interactions. The prevalence of infestation was calculated as the proportion of cats harbouring at least one flea, expressed as a percentage of the total number of cats examined. This parameter reflects the overall likelihood of flea presence within the sampled cat population. Host-related risk factors, including urban area type (housing, eatery, public transportation), cat sex, and age, were evaluated for their association with flea prevalence, following approaches commonly used in studies of flea-borne pathogens in cats [29,30,31].

Descriptive statistics were used to summarise flea infestation levels and *Bartonella* detection rates. Associations between categorical variables and flea infestation status were assessed using Fisher’s Exact Test. A *p*-value of <0.05 was considered statistically significant. All statistical analyses were performed using GraphPad Prism v9 (GraphPad Software, Boston, MA, USA).

## 3. Results

### 3.1. Flea Collection and Prevalence

A total of 204 fleas were recovered from 35 of the 89 stray cats sampled across various Klang Valley localities, resulting in an overall flea infestation prevalence of 39.33%. Fleas were detected at all surveyed locations except Kampung Baru. The highest flea burden originated from Pantai Dalam (*n* = 94; 45.19%), followed by Vista Angkasa Apartment (*n* = 53; 25.98%) and Kajang (*n* = 25; 12.25%) (Table 4 and Table S1).

Female cats comprised the majority of the sampled population (77.53%), and 27 of them (39%) were infested. Male cats represented a smaller portion of the sample (22.47%), but their infestation prevalence (40%) was almost identical to that of females (Table 5). This similarity suggests that flea exposure occurs uniformly across genders, likely driven by shared environmental conditions rather than sex-specific behaviour. Fisher’s Exact Test confirmed the absence of a statistically significant association between flea infestation and gender (*p* = 0.5711).

Adult cats showed an infestation prevalence of 39.0%, while juveniles showed a comparable prevalence of 40.0% (Table 5). No significant association was detected between infestation status and age class (*p* = 0.6571).

Environmental analysis revealed more pronounced differences. Flea infestation was recorded in all three major categories of sampling sites, namely housing, eatery, and public transportation areas. Public transportation areas exhibited the highest infestation prevalence (100%), indicating a consistently high exposure risk at these highly transient, human-associated locations. Housing areas showed a moderate prevalence of 40%, while eatery areas displayed the lowest prevalence at 33.9% (Table 5). Despite this lower prevalence, eatery zones contributed the largest number of fleas overall (*n* = 136), largely due to the high number of cats sampled.

### 3.2. Flea Identification

All 204 fleas were morphologically identified as *Ctenocephalides felis* (the cat flea) using established taxonomic keys [20]. Diagnostic characteristics included a long head with an angular frons, genal comb spines 1 and 2 of nearly equal length, and a distinct narrow dorsal incrassation [32]. No other flea species were detected among the examined specimens. For molecular confirmation, DNA from 118 individual fleas was successfully amplified targeting the mitochondrial *cox1* gene (~550 bp). Ten representative sequences (479 bp) showed 97–100% similarity with *C. felis* isolate Malay01 (GenBank accession No.: MT027230.1) in BLAST via the online NCBI platform, confirming morphological identification and demonstrating high sequence consservation among local populations (Table 6).

### 3.3. Molecular Detection of Bartonella spp.

Of the 118 flea DNA samples, 102 (86.44%) tested positive for *Bartonella* spp. via PCR targeting the *gltA* gene, producing amplicons of the expected 379 bp size (Table 2). Twelve representative positive samples from different locations were sequenced for species identification. BLAST analysis of the 266 bp partial *gltA* sequences revealed the presence of two recognized species, *B. henselae* and *B. claridgeiae*, along with two uncharacterized *Bartonella* strains (Table 7). Ten sequences exhibited ≥98% similarity with known type strains, confirming species identity. One sample (F103) displayed 92.51% similarity to *Bartonella* sp. isolate Dog_9 (GenBank accession No.: MN233800.1), suggesting a potentially novel or divergent strain, though still above the 95.4% similarity threshold for genus-level classification [33]. Specifically, three sequences (F01, F11, F33) matched *B. henselae* isolates from domestic cats in Brazil (MN107415.1), while seven (F23, F38, F44, F46, F83, F91, F109) were closely related to *B. claridgeiae* isolates PESET LAMADINUFF 23/25 (MH019300.1–MH019301.1). Another sample (F95) showed 98.48% identity with an uncultured *Bartonella* sp. clone (MH279890.1) from a crab-eating fox (*Cerdocyon thous*) in southern Brazil.

### 3.4. Phylogenetic Analysis of Fleas and Associated Bartonella spp.

**Figure 2:**
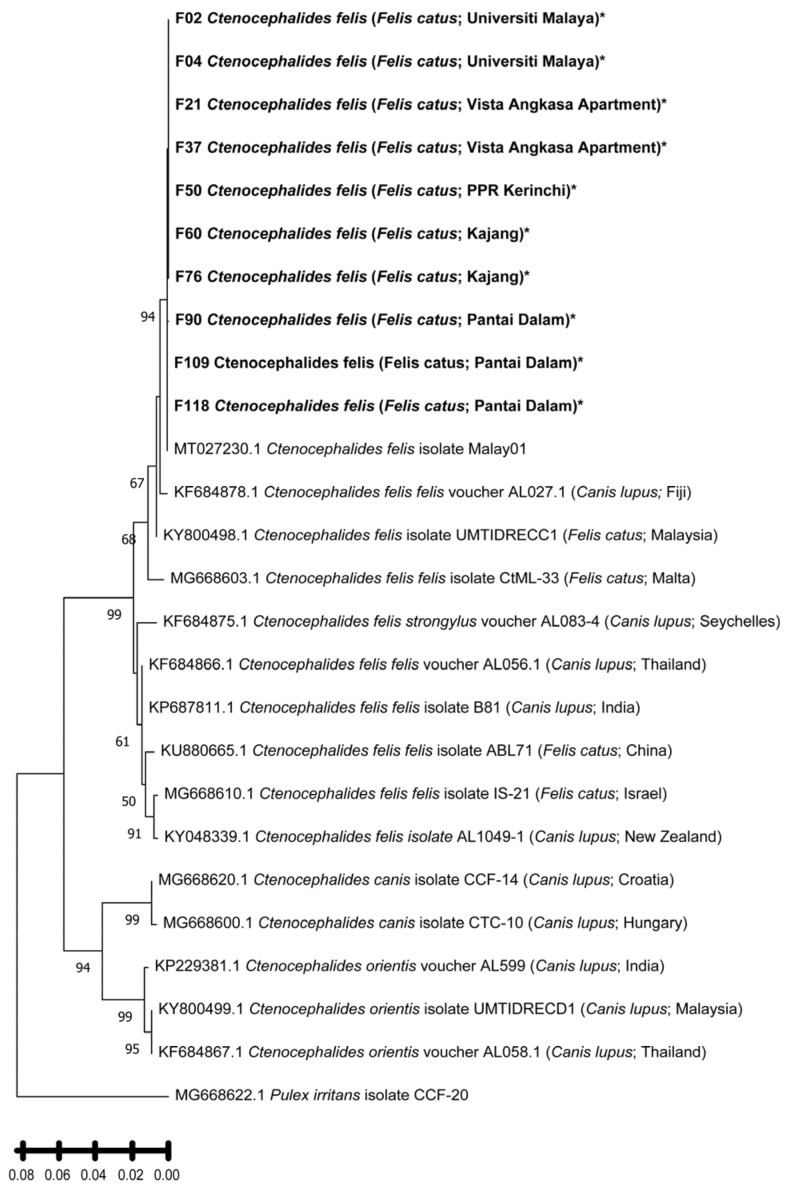
Phylogenetic tree based on 479 bp of *cox1* gene of flea sample from Klang Valley and constructed by using Molecular Evolutionary Genetics Analysis version 11 (MEGA11) software. The tree was obtained by using the Maximum Likelihood method and Tamura 3-parameter model. Bootstrap support was calculated by using 1000 replicates and >60% bootstrap values are shown. *Pulex irritans* isolate CCCF-20 was used as an outgroup for this study. Bold and asterisk (*) indicate the samples in this study.

**Figure 3:**
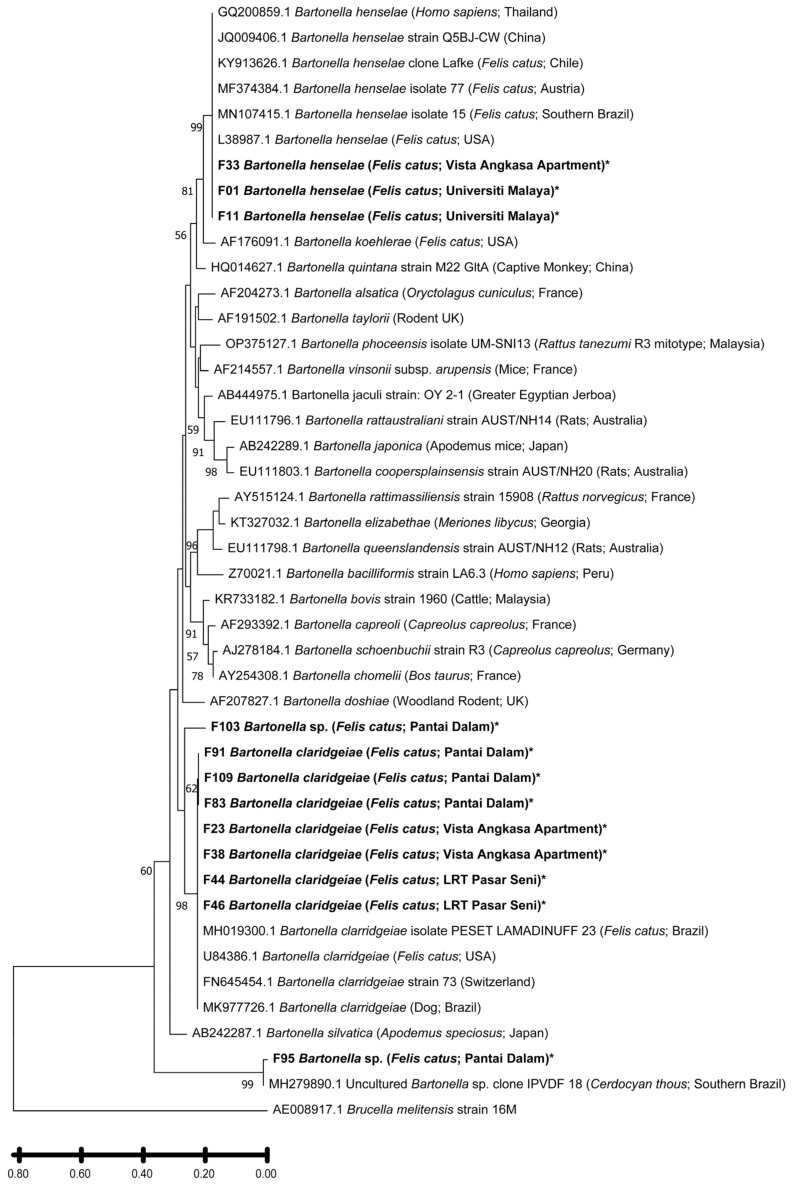
Phylogenetic tree based on 266 bp citrate synthase (*gltA*) gene of *Bartonella* sample from Klang Valley and constructed by using Molecular Evolutionary Genetics Analysis version 11 (MEGA11). The tree was obtained by using the Maximum Likelihood method and Kimura 2-parameter model. Bootstrap support was calculated by using 1000 replicates and >55% bootstrap values are shown. *Brucella melitensis* strain 16M was used as an outgroup for this study. Bold and asterisk (*) indicate the samples in this study.

Phylogenetic reconstruction of the *cox1* gene sequences confirmed that all flea samples obtained in this study belonged to *Ctenocephalides felis* (Figure 2). All sequences formed a strongly supported monophyletic cluster (bootstrap = 96%) together with *C. felis* isolate Malay01 (GenBank accession No.: MT027230.1), previously reported from cats in Southeast Asia. This cluster was clearly distinct from *Ctenocephalides canis* and *Ctenocephalides orientis*, which grouped into a separate, well-supported clade (bootstrap = 94%). The topology thus corroborates both the morphological and molecular identification of the fleas as *C. felis*. Phylogenetic analysis of the *Bartonella gltA* gene sequences revealed the presence of two well-defined clades, corresponding to *B. henselae* and *B. claridgeiae* (Figure 3). Sequences F01, F11, and F33 clustered tightly with *B. henselae* isolates previously reported from domestic cats in Brazil and the United States, supported by a bootstrap value of 99%. This *B. henselae* clade formed a sister relationship with *B. koehlerae* (GenBank accession No.: AF176091.1), indicating close evolutionary relatedness (bootstrap = 81%). The seven *B. claridgeiae* sequences (F23, F38, F44, F46, F83, F91, F109) formed a robust monophyletic clade (bootstrap = 98%) alongside reference strains from Brazil, the USA, and Switzerland. Notably, sequence F103, although related to this cluster, branched independently with relatively lower sequence similarity, suggesting possible intraspecific divergence or geographic variation. Sequence F95 was genetically distinct from both major clades but clustered closely with an uncultured *Bartonella* sp. clone (GenBank accession No.: MH279890.1) previously detected in a crab-eating fox (*Cerdocyon thous*) from Brazil (bootstrap = 99%). This unique positioning may reflect lineage diversification within *Bartonella* species infecting *C. felis* in Malaysia.

## 4. Discussion

This study provides updated insight into flea infestation patterns and *Bartonella* carriage among urban stray cats in the Klang Valley, a tropical city characterized by dense human–animal interactions and limited vector surveillance. The overall flea infestation rate of 39.3%, while lower than the national average of 71.8% reported by [30], remains epidemiologically significant and consistent with thresholds that warrant public health attention [34]. Comparable infestation levels have been reported in other urbanized tropical regions such as Mexico (53.0%) [35], though lower than observations from Thailand (95.8%) [36], Egypt (85.7%) [37], and Iran (92.3%) [38], likely reflecting differences in climate, host density, sampling approaches, and local veterinary practices.

Malaysia’s tropical climate, with high humidity and year-round warm temperatures, provides ideal conditions for continuous flea reproduction [39]. Stray cats in housing areas, eateries, and transportation hubs act as sentinels for urban flea persistence, highlighting the need for integrated ectoparasite surveillance within a One Health framework. Despite a limited sample size (*n* = 5) in transportation hubs, the observed 100% flea infestation prevalence is biologically plausible, consistent with reports of heavy ectoparasite burdens in unmanaged free-roaming cats in tropical urban environments [30]. High human mobility in these zones may further facilitate the dispersal of fleas or their developmental stages, enhancing the potential for silent vector transmission across the metropolitan area [27].

No significant associations were found between flea infestation and host sex or age, aligning with previous studies in Asia and the Middle East [36,37,38]. This suggests that exposure is driven more by environmental contact, grooming behaviors, and social interactions than by intrinsic host factors [40,41]. The predominance of female cats in the sampled population is likely a result of behavioral accessibility, as males often avoid humans due to territorial aggression, and should be considered in future sampling designs [30,42].

Morphological and molecular analyses confirmed *Ctenocephalides felis* as the sole flea species infesting stray cats in the Klang Valley, consistent with its global dominance among companion and stray animals [43,44,45]. COI barcoding enhanced taxonomic accuracy and confirmed close genetic similarity to the Southeast Asian isolate *C. felis* Malay01. The use of the mitochondrial COI marker is strongly supported by recent studies that demonstrate its reliability and resolution in distinguishing medically important flea species. For example, a 2025 study combining COI barcoding with scanning-electron microscopy (SEM) clearly differentiated *C. felis* from its close congener *Ctenocephalides orientis*, showing low intraspecific variation (0–0.24%) and sufficiently high interspecific divergence (4.6–21.3%) to support robust species delimitation [46]. Similarly, a large-scale survey characterizing the microbial communities of *C. felis* across the US and UK used *cox1* (COI) gene sequences to define flea haplotypes, and successfully correlated haplotype diversity with pathogen carriage, underlining COI’s utility in vector–pathogen ecology [47].

*Bartonella* DNA was detected in 86.4% of fleas, one of the highest prevalences reported worldwide, surpassing previous Malaysian (28.0%) [48], French (26.2%) [49], U.S. (22.8%) [50], and Palestinian (50.4%) [51] studies. Sequencing of the *gltA* gene identified two zoonotic species, *B. henselae* and *B. claridgeiae*, with *B. claridgeiae* predominating (58.3%), reflecting emerging trends in Asia and Latin America [52,53]. The co-circulation of these species underscores the dual role of stray cats and their fleas as reservoirs and amplifiers of zoonotic *Bartonella* transmission.

Notably, this study use of the citrate-synthase gene (*gltA*) alone for initial detection of *Bartonella* in fleas is consistent with long-standing and widely accepted practice in molecular epidemiology of *Bartonella*. Reviews of *Bartonella* genotyping show that *gltA* remains the most frequently used and reliable marker worldwide [54], especially in studies involving ectoparasites such as fleas, ticks, lice and bat flies [55]. In a comprehensive survey of 293 studies, *gltA*-based PCR (alone or with one additional locus) was used in nearly half of cases where one or two loci were employed, underscoring its central role in baseline detection [55]. Several large-scale field studies have successfully used *gltA*-only PCR to detect zoonotic *Bartonella* spp. in fleas. For instance, a survey of fleas collected from domestic animals across multiple bioclimatic zones in Tunisia screened over two thousand fleas and detected *Bartonella* DNA using *gltA* primers [44]. Similarly, in a study in France, fleas from cats were tested for *Bartonella* with *gltA* PCR, yielding meaningful prevalence data and species identification [49]. These examples demonstrate that *gltA* provides sufficient sensitivity and specificity to support epidemiological inference, particularly in preliminary surveillance studies. In recognition of current methodological advances, we acknowledge that multilocus PCR or next-generation sequencing (NGS) approaches (e.g., targeting *ssr*A, *rpo*B, ITS, *gro*EL) can enhance resolution, detect mixed infections, and improve phylogenetic depth [56]. Nevertheless, given resource constraints, the large number of flea specimens, and the primary aim of our study, to provide a baseline assessment of *Bartonella* presence in fleas from stray cats, using *gltA* as a first-line screening tool is justifiable and aligned with standard practice.

The detection of *Bartonella* DNA in fleas collected from free-roaming cats in urban Klang Valley raises significant public health concerns. The cat flea *Ctenocephalides felis* is a well-established vector for zoonotic *Bartonella* species (e.g., *B. henselae*, *B. claridgeiae*) and plays a central role in maintaining *Bartonella* transmission cycles among cats [57]. Molecular surveys from urban environments in East and Southeast Asia have documented the frequent presence of *Bartonella* in cat-infested fleas, highlighting their role as persistent reservoirs in anthropogenic settings [58]. In the Klang Valley, dense free-roaming cat populations in residential compounds, eateries, and public areas create frequent opportunities for human–cat interaction, including with vulnerable groups such as children and the elderly, facilitating accidental exposure to flea feces that can enter unnoticed abrasions or scratches. Bartonellosis is likely underdiagnosed in Malaysia due to limited clinical awareness and the absence of routine diagnostic testing such as PCR or serology in most hospitals [16,59,60]. While reported case numbers appear low, exposure risk is influenced more by the frequency of contact with infected vectors and animals than by formal notifications [61]. Although symptomatic disease is rare, zoonotic *Bartonella* infection can cause serious illness in immunocompromised or susceptible hosts [62]. Notably, the detection of divergent genotypes (*Bartonella* sp. clone IPVDF_18 and isolate Dog_9) suggests potential novel or regionally adapted lineages, possibly reflecting global dispersal via companion animals or vector migration [63,64]. Taken together, these findings underscore the emerging risk of flea-borne *Bartonella* transmission in tropical urban settings and highlight the need for targeted surveillance, public awareness, and vector-control strategies to protect human health.

The coexistence of high *C. felis* infestation and elevated *Bartonella* infection highlights the emerging risk of flea-borne zoonoses in tropical urban settings. Stray cats serve as both reservoirs and amplifiers of vector populations capable of transmitting cat scratch disease to humans. The absence of systematic vector control and animal health monitoring facilitates ongoing pathogen circulation. Integrating flea surveillance with urban public health strategies, particularly through collaboration with municipal authorities and animal welfare organizations, could help mitigate zoonotic risks, in alignment with global One Health recommendations from WHO and OIE/WOAH [65,66].

## 5. Study Limitations

This study provides important baseline data on flea infestation and *Bartonella* transmission in urban stray cats, but several limitations exist. Sampling was limited to select urban sites in the Klang Valley, and broader geographic coverage, including peri-urban and rural areas, would better capture ecological variation. Molecular analysis relied on a single *gltA* locus, which, while widely used, has limited resolution for closely related *Bartonella* strains; multilocus or genomic approaches could improve species discrimination and detect mixed infections. The study focused solely on cats, though dogs, rodents, and other synanthropic wildlife may also serve as reservoirs of flea-borne pathogens. Finally, only *Bartonella* spp. were assessed, despite *C. felis* harboring multiple zoonotic agents, highlighting the need for integrated multi-pathogen surveillance to fully evaluate public health risks.

## 6. Conclusions

This study presents updated molecular and phylogenetic evidence of *Ctenocephalides felis*–borne *Bartonella* species circulating among stray cats in urban areas of the Klang Valley, Malaysia. The high prevalence of *C. felis* infestations and the notable detection rate of *Bartonella* DNA highlight a potentially underestimated zoonotic risk within densely populated urban environments. Phylogenetic analyses based on *cox1* and *gltA* genes confirmed that all flea specimens were *C. felis* and identified *B. henselae* and *B. claridgeiae*, with several genetically diverse lineages suggesting possible novel or geographically adapted strains. These findings emphasise the importance of ongoing molecular surveillance of flea-borne pathogens in stray and domestic animal populations, especially in tropical cities where close human–animal contact facilitates transmission. Integrated control strategies combining vector management, stray animal health programmes, and public awareness are crucial to reducing the risk of *Bartonella* and other flea-borne pathogens spilling over to humans. Future research employing multi-locus or whole-genome approaches will further clarify the evolutionary dynamics and public health implications of these emerging pathogens in Southeast Asia.

## Figures and Tables

**Table 1:** The sampling sites visited for flea sampling within Klang Valley, Peninsular Malaysia.

Location Type	Location Code	Location Name	Coordinate
Housing	Site 1	Vista Angkasa Apartment	3.113557556852751, 101.66219419555847
	Site 2	PPR Kerinchi	3.10639539204193, 101.66874288244868
	Site 3	Pangsapuri 17 Tingkat Kerinchi	3.112143440092739, 101.66271990854636
Eatery	Site 4	Universiti Malaya	3.1219803614182364, 101.65692982254491
	Site 5	Kampung Baru	3.1622532764570286, 101.70469742056846
	Site 6	Kajang	2.98559539628367, 101.78108597466827
	Site 7	Pantai Dalam	3.088280610413649, 101.66833646527007
Public Transportation	Site 8	LRT Universiti Station	3.1147193277270824, 101.66170419932773
	Site 9	LRT Kerinchi Station	3.115756971841698, 101.668254108292
	Site 10	LRT Pasar Seni Station	3.1423221347431975, 101.69552125239596

**Table 2:** Primers used for PCR assays in this study.

Gene	Primer Name	Sequence (5′-3′)	Direction	Detection	Reference
*cox1*	LCO	GGT CAA CAA ATC ATA AAG ATA TTG G	Forward	Flea species	[9]
	Cff-R	GAA GGG TCA AAG AAT GAT GT	Reverse		
*gltA*	BhCS.781p	GGG GAC CAG CTC ATG GTG G	Forward	*Bartonella*	[10]
	BhCS.1137n	AAT GCA AAA AGA ACA GTA AAC A	Reverse		

**Table 3:** Cycling conditions used for PCR assays in this study.

Gene	Initial Denaturation	Denaturation	Annealing	Extension	Final Extension	Cycle	Amplicon Size (bp)	Reference
*cox1*	95 °C 1 min	95 °C 15 s	55 °C 15 s	72 °C 10 s	72 °C 5 min	35	550	[9]
*gltA*	94 °C 10 min	94 °C 30 s	51 °C 45 s	72 °C 30 s	72 °C 7 min	35	379	[10]

**Table 4:** Flea infestation in free-roaming cats across different urban locations in the Klang Valley, Malaysia.

Location Code	Number of Cats Examined	Number of Cats Infested with Fleas (Prevalence, %)	Total Fleas Collected
Site 1	14	7 (50.0)	53
Site 2	7	1 (14.2)	3
Site 3	4	2 (50.0)	3
Site 4	26	6 (23.1)	17
Site 5	7	0 (0.0)	0
Site 6	5	3 (60.0)	25
Site 7	21	11 (52.4)	94
Site 8	2	2 (100.0)	2
Site 9	1	1 (100.0)	3
Site 10	2	2 (100.0)	4
Total	89	35	204

**Table 5:** Prevalence of fleas (*n* = 204) on 89 sampled cats based on identified risk factors in the study areas.

Risk Factor	Variable	Flea-Infested Cat	Total Fleas	Prevalence of Infestation (%)
Area	Housing area (*n* = 25)	10	59	40.0
	Eatery area (*n* = 59)	20	136	33.9
	Public transportation area (*n* = 5)	5	9	100.0
Gender	Male (*n* = 20)	8	35	40.0
	Female (*n* = 69)	27	169	39.0
Age	Adult (*n* = 84)	33	200	39.0
	Juvenile (*n* = 5)	2	4	40.0

Note: Prevalence of infestation was calculated as the proportion of cats harbouring at least one flea, expressed as a percentage of the total number of cats examined.

**Table 6:** BLASTn results for the obtained flea sequences based on the *cox1* gene region.

Flea in This Study	Location	Closest GenBank Match	Maximum Identity (%)	Query Cover (%)	E-Value	Origin
F02	University Malaya	*C. felis* isolate Malay01 (MT027230.1)	99.83	99	0.0	East and Southeast Asia
F04	University Malaya	*C. felis* isolate Malay01 (MT027230.1)	97.83	98	0.0	East and Southeast Asia
F21	Vista Angkasa Apartment	*C. felis* isolate Malay01 (MT027230.1)	100.00	98	0.0	East and Southeast Asia
F37	Vista Angkasa Apartment	*C. felis* isolate Malay01 (MT027230.1)	100.00	98	0.0	East and Southeast Asia
F50	PPR Kerinchi	*C. felis* isolate Malay01 (MT027230.1)	100.00	100	0.0	East and Southeast Asia
F60	Kajang	*C. felis* isolate Malay01 (MT027230.1)	100.00	100	0.0	East and Southeast Asia
F76	Kajang	*C. felis* isolate Malay01 (MT027230.1)	100.00	98	0.0	East and Southeast Asia
F90	Pantai Dalam	*C. felis* isolate Malay01 (MT027230.1)	100.00	100	0.0	East and Southeast Asia
F109	Pantai Dalam	*C. felis* isolate Malay01 (MT027230.1)	100.00	100	0.0	East and Southeast Asia
F118	Pantai Dalam	*C. felis* isolate Malay01 (MT027230.1)	99.83	98	0.0	East and Southeast Asia

**Table 7:** The BLASTn results for the obtained *Bartonella* sequences based on *gltA* gene region.

Sample ID	Location	Closest GenBank Match	Maximum Identity (%)	Query Cover (%)	E-Value	Origin
F01	University Malaya	*B. henselae* isolate 15 (MN107415.1)	99.70	100	5 × 10^−172^	Brazil
F11	University Malaya	*B. henselae* isolate 15 (MN107415.1)	99.70	100	5 × 10^−172^	Brazil
F23	Vista Angkasa Apartment	*B. claridgeiae* isolate PESET LAMADINUFF 23 (MH019300.1)	99.41	100	2 × 10^−170^	Brazil
F33	Vista Angkasa Apartment	*B. henselae* isolate 15 (MN107415.1)	98.53	100	2 × 10^−166^	Brazil
F38	Vista Angkasa Apartment	*B. claridgeiae* isolate PESET LAMADINUFF 23 (MH019300.1)	99.41	100	2 × 10^−170^	Brazil
F44	LRT Pasar Seni	*B. claridgeiae* isolate PESET LAMADINUFF 25 (MH019301.1)	99.69	96	6 × 10^−166^	Brazil
F46	LRT Pasar Seni	*B. claridgeiae* isolate PESET LAMADINUFF 23 (MH019300.1)	99.70	100	2 × 10^−171^	Brazil
F83	Pantai Dalam	*B. claridgeiae* isolate PESET LAMADINUFF 25 (MH019301.1)	99.11	100	1 × 10^−167^	Brazil
F91	Pantai Dalam	*B. claridgeiae* isolate PESET LAMADINUFF 25 (MH019301.1)	98.24	100	1 × 10^−163^	Brazil
F95	Pantai Dalam	Uncultured *Bartonella* sp. clone IPVDF_18 (MH279890.1)	98.48	98	2 × 10^−160^	Brazil
F103	Pantai Dalam	*Bartonella* sp. isolate Dog_9 (MN233800.1)	92.51	99	4 × 10^−128^	Chile
F109	Pantai Dalam	*B. claridgeiae* isolate PESET LAMADINUFF 23 (MH019300.1)	99.41	100	8 × 10^−170^	Brazil

## Data Availability

The original contributions presented in this study are included in the article/Supplementary Material. Further inquiries can be directed to the corresponding authors.

## Supplementary Materials

The following supporting information can be downloaded at: https://www.mdpi.com/article/10.3390/insects16121282/s1, Table S1: The number of fleas collected by hosts within Klang Valley area.
