# Supplementary material for: Emerging Risk of Flea-Borne Bartonella in Tropical Cities: Evidence from Stray Cats in the Klang Valley, Malaysia
Source: Insects. 2025 Dec 18;16(12):1282. doi: 10.3390/insects16121282 (PMC12733989; doi:10.3390/insects16121282)
Supplement: Supplementary file 1 [file insects-16-01282-s001.zip › Table S1.pdf]

Table S1: The number of fleas collected by host within Klang Valley area.

Location	Date of Sampling	Host ID	Sex (Male/Female)	Age (Adult/Juvenile)	Number of Fleas Collected
**University Malaya**	18/10/2023	UM-01	Female	Adult	0
		UM-02	Female	Adult	0
		UM-03	Female	Adult	0
		UM-04	Female	Adult	0
		UM-05	Male	Adult	1
		UM-06	Female	Adult	2
		UM-07	Female	Adult	11
		UM-08	Female	Juvenile	1
		UM-09	Male	Adult	0
		UM-10	Male	Adult	1
	24/10/2023	UM-11	Female	Adult	0
		UM-12	Male	Adult	0
		UM-13	Female	Adult	0
		UM-14	Female	Adult	0
		UM15	Female	Adult	0
	25/10/2023	UM-16	Female	Adult	0
		UM-17	Male	Adult	0
		UM-18	Male	Adult	0

		UM-19	Female	Adult	0
		UM-20	Male	Adult	0
		UM-21	Female	Adult	0
		UM-22	Male	Adult	1
		UM-23	Female	Adult	0
		UM-24	Female	Juvenile	0
		UM-25	Male	Juvenile	0
		UM-26	Female	Adult	0
**Vista Angkasa Apartment**	8/11/2023	VA-01	Female	Adult	0
		VA-02	Female	Adult	4
		VA-03	Female	Adult	0
		VA-04	Female	Adult	0
		VA-05	Female	Adult	0
		VA-06	Female	Adult	0
		VA-07	Female	Adult	19
		VA-08	Female	Adult	0
		VA-09	Female	Adult	1
	13/11/2023	VA-10	Male	Adult	23
		VA-11	Male	Adult	2
		VA-12	Female	Adult	0
		VA-13	Female	Adult	3

		VA-14	Female	Adult	1
**LRT Universiti**	20/11/2023	LRT-01	Female	Adult	1
		LRT-02	Female	Adult	1
**LRT Kerinchi**		LRT-03	Male	Adult	3
**LRT Pasar Seni**		LRT-04	Female	Adult	2
		LRT-05	Female	Adult	2
**Kampung Baru**	27/11/2023	KB-01	Female	Adult	0
		KB-02	Female	Adult	0
		KB-03	Male	Adult	0
		KB-04	Female	Adult	0
		KB-05	Male	Adult	0
		KB-06	Female	Adult	0
		KB-07	Female	Adult	0
**PPR Kerinchi**	15/12/2023	PPR-01	Female	Adult	3
		PPR-02	Male	Adult	0
		PPR-03	Female	Adult	0
		PPR-04	Female	Adult	0
		PPR-05	Female	Adult	0
		PPR-06	Male	Adult	0
		PPR-07	Female	Adult	0

**Pangsapuri 17 Tingkat Kerinchi**		PTK-01	Female	Adult	2
		PTK-02	Female	Adult	1
		PTK-03	Female	Adult	0
		PTK-04	Female	Adult	0
**Kajang**	4/1/2023	KJ-01	Female	Adult	0
		KJ-02	Female	Adult	10
		KJ-03	Female	Adult	10
		KJ-04	Male	Adult	0
		KJ-05	Female	Adult	5
**Pantai Dalam**	4/1/2023	PD-01	Female	Adult	0
		PD-02	Female	Adult	0
		PD-03	Female	Adult	0
		PD-04	Male	Adult	2
		PD-05	Female	Adult	4
		PD-06	Female	Adult	0
		PD-07	Female	Adult	1
		PD-08	Female	Adult	1
		PD-09	Female	Adult	0
		PD-10	Female	Adult	0
		PD-11	Female	Adult	51
		PD-12	Male	Adult	2

	PD-13	Female	Adult	0
	PD-14	Female	Adult	0
	PD-15	Female	Adult	1
	PD-16	Female	Adult	0
	PD-17	Female	Adult	17
	PD-18	Female	Juvenile	3
	PD-19	Female	Juvenile	0
	PD-20	Female	Adult	3
	PD-21	Female	Adult	9
